# The Use of Stable and Radioactive Sterol Tracers as a Tool to Investigate Cholesterol Degradation to Bile Acids in Humans *in Vivo*

**DOI:** 10.3390/molecules17021939

**Published:** 2012-02-16

**Authors:** Marco Bertolotti, Andrea Crosignani, Marina Del Puppo

**Affiliations:** 1 Divisone di Geriatria, Dipartimento di Medicina, Endocrinologia, Metabolismo e Geriatria, Università degli Studi di Modena e Reggio Emilia, Nuovo Ospedale Civile, Via Giardini 1355, Modena 41126, Italy; 2 Dipartimento di Medicina Interna, Epatologia e Gastroenterologia, Università degli Studi di Milano, Ospedale San Paolo, Via Di Rudinì 8, Milano 20142, Italy; 3 Dipartimento di Medicina Sperimentale, Università degli Studi di Milano Bicocca, Via Cadore 48, Monza 20052, Italy

**Keywords:** bile acid kinetics, cholesterol degradation, human studies *in vivo*, sterol tracers

## Abstract

Alterations of cholesterol homeostasis represent important risk factors for atherosclerosis and cardiovascular disease. Different clinical-experimental approaches have been devised to study the metabolism of cholesterol and particularly the synthesis of bile acids, its main catabolic products. Most evidence in humans has derived from studies utilizing the administration of labeled sterols; these have several advantages over *in vitro* assay of enzyme activity and expression, requiring an invasive procedure such as a liver biopsy, or the determination of fecal sterols, which is cumbersome and not commonly available. Pioneering evidence with administration of radioactive sterol derivatives has allowed to characterize the alterations of cholesterol metabolism and degradation in different situations, including spontaneous disease conditions, aging, and drug treatment. Along with the classical isotope dilution methodology, other approaches were proposed, among which isotope release following radioactive substrate administration. More recently, stable isotope studies have allowed to overcome radioactivity exposure. Isotope enrichment studies during tracer infusion has allowed to characterize changes in the degradation of cholesterol via the “classical” and the “alternative” pathways of bile acid synthesis. Evidence brought by tracer studies *in vivo*, summarized here, provides an exceptional tool for the investigation of sterol metabolism, and integrate the studies *in vitro* on human tissue.

## Abbreviations

CA: cholic acid
CDCA: chenodeoxycholic acid
CYP7A1: cholesterol 7α-hydroxylase
CYP27A1: sterol 27-hydroxylase
CTX: cerebrotendinous xanthomatosis
DCA: deoxycholic acid
FGF: fibroblast growth factor
FXR: farnesoid X receptor
LXR: liver X receptor
PPAR: peroxysomal proliferator-activated receptor
SHP: small heterodimer partner
SREBP: sterol regulatory element binding protein
UDCA: ursodeoxycholic acid

## 1. Introduction

Cholesterol homeostasis in the whole organism is regulated by a complex series of interrelated metabolic pathways. Whenever such homeostatic balance is altered, an accumulation of cholesterol in one or more body compartments may take place [1], with the possible occurrence of clinically relevant cholesterol accumulation conditions, such as atherosclerosis or cholesterol gallstone disease. In this context, the liver plays a key role [2]. In the liver a delicate balance exists between input and output pathways [3], so that under physiological conditions no net accumulation of this sterol takes place.

Degradation to primary bile acids represents the most relevant mechanism of irreversible elimination of cholesterol from the body, thus playing a key role in hepatic and systemic cholesterol homeostasis. Under physiological conditions, approximately 300–400 mg of cholesterol are disposed of in the liver daily, through this pathway [3].

Alterations in bile acids production can therefore have relevant consequences on the cholesterol content in different body compartments, as exemplified by the clinical-experimental model of the treatment with bile acid-binding resins, which are capable of reducing plasma cholesterol levels by increasing bile acid production [4].

Over the past two decades and with the aid of molecular biology a body of evidence has defined the molecular mechanisms of the regulation of cholesterol and bile acid metabolism; among others, the role of nuclear receptors in the control of bile acid synthesis and of the activity of cholesterol 7α-hydroxylase, the limiting enzyme of the “classical” biosynthetic pathway, has been highlighted [3,5,6,7,8]. Furthermore, the role of the so-called “alternative” pathway of bile acid synthesis, whose first step is hepatic and extrahepatic 27-hydroxylation of cholesterol, has been underlined [9,10,11].

Different experimental approaches have been devised to study bile acid metabolism. Together with the analytical assay of enzyme expression and activity in human tissue *in vitro*, a large amount of evidence has been obtained *in vivo* in humans with the aid of administration of labeled sterols.

This review will summarize the main evidence coming from human studies with the use of radioactive or stable isotopes as metabolic tracers; such evidence has provided an extraordinary contribution to our knowledge on the regulation of cholesterol degradation to bile acids, both via the “classical” and the “alternative” pathways of bile acid synthesis.

## 2. Biochemistry of Bile Acid Synthesis

Bile acids are final water-soluble products of cholesterol catabolism and their synthesis represents a relevant metabolic step in the regulation of whole body cholesterol balance. Bile acids are 24 carbon atom steroidal carboxylic acids derived from cholesterol. The primary bile acids in humans, cholic acid (CA) and chenodeoxycholic acid (CDCA), are synthesized in the liver and conjugated with taurine or glycine before secretion via bile into the intestine. The cholesterol conversion into CA and CDCA occurs prevalently in hepatocytes and involves different steps: the initiation of synthesis by hydroxylation of cholesterol, further modifications to the ring structures, side-chain oxidation and shortening by three carbons, and conjugation of the bile acid with taurine or glycine [12].

Two main pathways of bile acid synthesis have been described [10]. The classical “neutral” pathway starts with 7α-hydroxylation of cholesterol and is catalyzed by the liver-specific enzyme cholesterol 7α-hydroxylase (CYP7A1). This enzyme, a specific microsomal cytochrome P450 expressed only in the liver, represents the rate-limiting step. CYP7A1 is reported to be active almost exclusively on cholesterol and cholestanol, its 5α-saturated analog [13], thus showing a limited substrate specificity. Nevertheless other authors reported that this enzyme is able to catalyze 7α-hydroxylation also of 27-hydroxycholesterol and other oxysterols [14].

Bile acids returning to the liver through the enterohepatic circulation were considered as the effectors of a feedback control on the biosynthetic pathway [4,15,16]. Bile acid feedback inhibition of CYP7A1 is mediated, at least in experimental models, by the nuclear receptors farnesoid X receptor (FXR) and small heterodimer partner (SHP) [17]. Experimental data demonstrate that bile acids block the association of the coactivators peroxysomal proliferator-activated receptor (PPAR) gamma coactivator-1α (PGC-1α) and cAMP Response Element Binding protein-Binding Protein (CBP) with Hepatocyte Nuclear Factor-4α (HNF-4α) and suppress the transcription of CYP7A1 in an FXR-independent manner [18].

The 7α-hydroxycholesterol is next converted into 7α-hydroxy-4-cholesten-3-one by a microsomal 3β-hydroxy-Δ^5^-C27-steroid dehydrogenase/oxidoreductase (C_27_β-HSD) located in the endoplasmic reticulum which catalyzes the isomerization of the double bond and oxidation of the hydroxyl group in position 3 to an oxo group. Next, 7α-hydroxy-4-cholesten-3-one is converted to 7α,12α-dihydroxy-4-cholesten-3-one by a sterol 12α-hydroxylase a microsomal cytochrome P-450 (CYP8B1) [19].

Further reduction of the 7α-hydroxy-4-cholesten-3-one and 7α,12α-dihydroxy-4-cholesten-3-one catalyzed by Δ^4^-3-oxosteroid-5β-reductase and 3α-hydroxysteroid dehydrogenase [20,21] produce the precursors of CDCA and CA (5β-cholestane-3α,7α-diol and 5β-cholestane-3α,7α,12α-triol, respectively).

The subsequent side-chain oxidation of diol and triol steroids by mitochondrial sterol 27-hydroxylase (CYP27A1) and cleavage of C_24_-C_25_ bond are the next steps in the biosynthetic pathway leading to primary bile acid synthesis. In humans the classical pathway is the most relevant one in quantitative terms, in normal condition and produces CA and CDCA acid in roughly equal quantities.

Bile acid synthesis occurs also via an “alternative” pathway for which the first step, which precedes the modifications to the ring nucleus, is represented by the hydroxylation of the cholesterol side chain at position 27 catalyzed by CYP27A1 [9,10], a mitochondrial cytochrome P-450 characterized by broad substrate specificity and by broad tissue and organ distribution, in particular in vascular endothelium, in fibroblasts and in macrophages [9]. Further steps in bile acid synthesis via this pathway include oxidation of 27-hydroxycholesterol to cholestenoic acid catalyzed by the same enzyme [22,23]. In this way sterol 27-hydroxylase promotes metabolism of cholesterol to more polar compounds that are more efficiently exported from cells than the parent compound [24] and transported through the circulation to the hepatic cells where hydroxylation at position 7α by oxysterol 7α-hydroxylase (CYP7B1) [25] and the side chain cleavage rise particularly to CDCA. 

The role of the alternative biosynthetic pathway in quantitative terms is controversial: in human subjects, bile acid production via 27-hydroxylation accounts only for 10% of total bile acid synthesis [26]. Nevertheless the alternative pathway could represent an important means for removal of cholesterol deposited in endothelium and it is considered to be important in the reverse transport of cholesterol, from the periphery to the liver and its protective role against atherosclerosis has been proposed [27]. 

*In vivo* conversion of cholesterol into bile acids occurs also following two quantitatively minor pathways involving introduction of one hydroxyl group at two different positions of side chain of cholesterol C24S and C25 (24- and 25-hydroxylase pathways). 

**Scheme 1:**
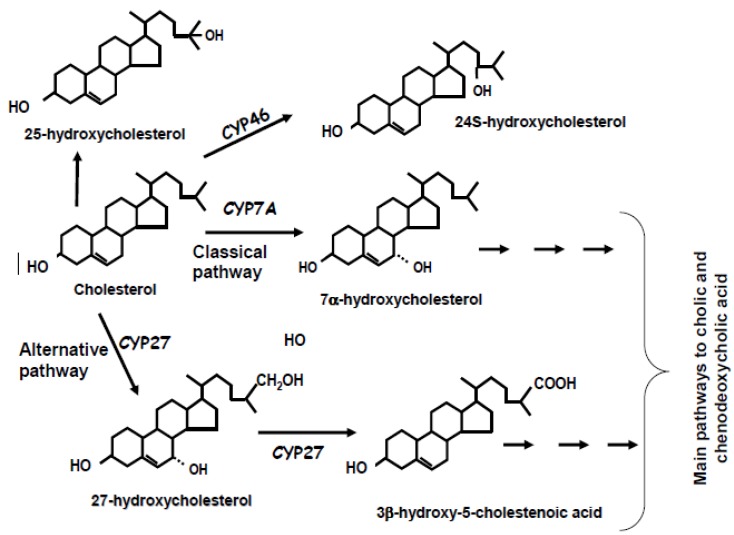
Schematic illustration of the main pathways of bile acid synthesis from cholesterol.

**Scheme 2:**
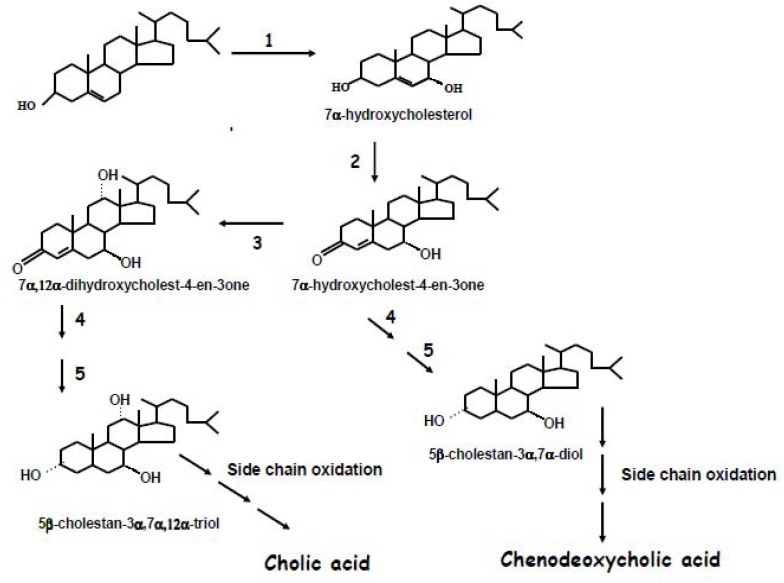
Illustration of the main metabolic steps of the classical pathway of bile acid synthesis. **1**: Cholesterol-7α-hydroxylase (CYP7A); **2**: 3β-hydroxy-Δ^5^-C27-steroid dehydrogenase (C_27_β-HSD); **3**: Sterol 12α-hydroxylase (CYP8B1); **4**: Δ^4^-3-oxosteroid-5β-reductase; **5**: 3α-hydroxysteroid dehydrogenase.

The 24-hydroxylase pathway in particular allows maintenance of cholesterol homeostasis in brain by conversion into 24*S*-hydroxycholesterol, also known as cerebrosterol. This reaction is catalyzed by the cholesterol 24-hydroxylase (CYP46) [28]. 24S-Hydroxycholesterol is more polar than cholesterol and it is able to pass the lipophilic membranes, such as the blood-brain barrier for transport through the circulation to the liver and further conversion to bile acids. Scheme 1 and Scheme 2 illustrate in a schematic way the main steps involved in the degradation of cholesterol to bile acids.

## 3. Alternative Approaches for the Quantification of Bile Acid Synthesis

Extensive review of the different procedures and techniques adopted in the past and at the present time to measure bile acid synthesis is way beyond the aims of the present paper. Only a brief mention will be made to alternative techniques such as *in vitro* assays, fecal sterol analysis, and determination of plasma concentrations of non-cholesterol sterols.

The determination of bile acid synthesis and the study of the alterations induced by disease conditions and by treatment have long relied upon the assay *in vitro* of the expression and activity of the limiting enzyme, CYP7A1, and, more recently, of CYP27A1. 

Since the 1960s pioneering evidence has been obtained with the assay of CYP7A1 activity after isolation of liver microsomes [29,30]. Such assays have utilized radioactive isotopes and, in more recent years, stable tracers for the determination of CYP7A1 activity. Using these techniques, results of paramount importance have been obtained in the field of the physiology and pathophysiology of the regulation of bile acid synthesis, including the relevance of the physical-chemical properties of bile acids, often replicating in humans the results obtained in animal models [3,15,19]. Evidence on the role of 27-hydroxylase has emerged as well over the past decades [9,10], even if direct studies in human subjects have been extremely scarce in this regard, despite the fact that CYP27A1 is expressed also in macrophages, which are easily obtainable from circulating blood, after isolation of monocytes and *in vitro* monocyte/macrophage differentiation [31].

More recently, translational research has been extended to the assay of tissue gene expression (mRNA) and protein content, for both CYP7A1 and CYP27A1 [3,7,8].

An obvious limit of all *in vitro* procedures is the need to obtain tissue (usually liver) specimens; these can usually be obtained during surgical procedures, or by means of non-surgical biopsy. The relative invasiveness and inherent ethical problems clearly represent a major drawback with this kind of approach.

Analysis of fecal sterols has extensively been utilized, in the attempt to overcome the need of *ex vivo* tissue samples. The measurement of fecal acidic sterols can provide an indirect measurement of whole body synthetic rates [32,33,34]. This has been tested and validated in several experimental situations [35,36].

This approach too has yielded important results in the knowledge of the regulation of bile acid synthesis. Nonetheless, it requires accurate fecal collection and the techniques for the analytical determinations are usually rather complex, making this approach not suitable for wide scale utilization.

Finally, the determination of plasma or serum levels of hydroxylated sterols which represent metabolic intermediates in the biosynthetic pathway has been extensively utilized as an indirect measure of bile acid synthesis or of some of its rate-limiting steps. 

The assay of circulating levels of 7α-hydroxycholesterol, the end-product of the reaction catalyzed by CYP7A1, has been proposed for long time as an indirect measurement of bile acid synthesis and of CYP7A1 activity, particularly in stimulated conditions [37,38]. Analysis of the more stable metabolite 7α-hydroxy-4-cholesten-3-one (C4) has also been used as a surrogate marker of bile acid synthesis rates [39,40,41,42].

Similarly, the determination of serum levels of 27-hydroxycholesterol has been used as an index of hepatic and extrahepatic CYP27 activity, and therefore of the initial steps in the alternate pathway of bile acid synthesis [31,43,44,45]. 

The simplicity and convenience of similar approaches, requiring a single blood sample, makes them ideal for large-scale investigation, if not for routine analysis. We need to keep in mind, though, that this kind of determination can only provide semi-quantitative estimates of bile acid synthesis and therefore cannot be utilized in the evaluation of homeostatic changes of cholesterol and bile acid metabolism.

## 4. Use of Radioactive Tracers for the Measurement of Bile Acid Synthesis

The gold standard technique for the determination of total bile acid synthesis in humans *in vivo* has been represented, over the past half-century, by the isotope dilution method, in origin described by Lindstedt in the late 50s [46]. Such a technique is based on the principle that bile acid turnover undergoes first order kinetics and therefore, after labeled bile acid administration, their specific radioactivity decreases monoexponentially with time. According to this approach, oral administration of radioactive derivatives of bile acids, usually cholic acid (CA) and chenodeoxycholic acid (CDCA) labeled with either [^14^C] or [^3^H], is followed by serial duodenal bile sampling over the following days [46,47].

Liquid scintillation analysis of the radioactivity in bile samples, usually following bile acid extraction and separation, allows determination of turnover rate, pool size and synthetic rate of primary bile acids.

This technique is relatively simple and non-expensive; it has the inconvenience of the need for repeated bile sampling, which is usually obtained by duodenal intubation, a procedure not well tolerated by most patients.

A simplification of such techniques has been later proposed [48] consisting in administration in different time points of CA and CDCA labeled with different tracers followed by a single bile collection, thus minimizing the inconvenience of duodenal intubation. Multi-compartmental model analysis was also adopted to define the kinetic parameters of administered bile acids [49].

Combined administration of radiolabeled tracers can allow selective estimation of the relative contributions of the “classical” and “alternate” pathways of bile acid synthesis, as more recently proposed by Duane and Javitt [50]. Administration of [^14^C]-labeled CA and CDCA together with [7β -^3^H]7α(OH)cholesterol or [22,23-^3^H]27(OH)cholesterol was used, allowing to calculate the production rates of primary bile acids by classical isotope dilution technique, and to estimate the relative contribution of the two precursors from the [^3^H/^14^C] ratio in CA and CDCA.

Alternative procedures to isotope dilution have also been proposed with the approach of isotope release. The technique described by Rosenfeld *et al.* [51,52] is based upon i.v. administration of [24,25-^3^H]cholesterol. After side chain cleavage of the cholesterol molecule, [^3^H] release is proportional to, and can provide an estimate of, bile acid synthesis rate. This can be calculated as the ratio between body water tritium enrichment, assayed after distillation of red blood cells, and the specific activity of plasma cholesterol in plasma.

A similar approach has been described by Duane and coworkers with the measurement of [^14^C]CO_2_ release after i.v. administration of [26-^14^C]cholesterol; here, like in the previous approach, the technique can estimate bile acid synthesis by determination of side chain cleavage. According to this methodology, the output of [^14^C]CO_2_ is collected from the breath at time intervals after tracer administration, with the aid of an air-trapping device [53]. The rate of bile acid synthesis can be calculated as the ratio between the output of [^14^C]CO_2_ and the specific radioactivity of plasma cholesterol, measured in the same time frame. This procedure was also described to detect short-term changes of bile acid synthesis rates, as occurring after bolus drug administration [54]. A disadvantage of this approach is the requirement for a dedicated breath-collecting apparatus.

Another isotope release approach has been developed by our group over the past three decades: this approach is based upon the principle that cholesterol 7α-hydroxylation, the first and rate-limiting step of the biosynthetic pathway, is a highly stereospecific reaction whereby a tritium atom at the 7α position is replaced by a hydroxyl group. After i.v. administration of a bolus of [7α-^3^H]cholesterol the amount of tritium exchanged will reflect the extent of the 7α-hydroxylation reaction and therefore the rate of bile acid synthesis. The latter can be directly calculated as the ratio between tritium enrichment in the body water pool (determined after distillation of biologic fluids, either blood or urine) and the specific radioactivity of serum cholesterol, analyzed in the same time period [55]. Because tritium release from the 7α position is independent of the specific enzymatic reaction involved, this assay can detect the amount of cholesterol degraded both via the classical and the alternate pathways.

This technique has proven extremely useful in detecting changes in bile acid synthesis in different clinical-experimental conditions (see later) and has been shown to correlate well with the assay of serum levels of 7α-hydroxy-4-cholesten-3-one, as an indirect measure of bile acid synthesis and of CYP7A1 activity [42].

A relative drawback with this technique is the need to obtain sufficient amounts of [7α-^3^H]cholesterol with a high degree of specific localization of the radiolabel at the 7α-position.

## 5. Use of Stable Isotopes for the Measurement of Bile Acid Synthesis

### 5.1. Bile Acid Kinetics Studies

All of the aforementioned techniques share the inconvenience of the systemic exposure of patients to ionizing radiations. Even if this can be theoretically quantified, and the amount of radioactivity absorbed was usually estimated not to exceed that of a routine radiological procedure [55], the risk of tissue damage from radioactivity has to be taken carefully into account, together with the inherent ethical concerns. The use of stable isotopes should theoretically overcome these safety concerns and indeed over the last decades a number of techniques have been developed with these compounds.

Since the 1970s, different research groups have described isotope dilution approaches, closely resembling that described earlier by Lindstedt, utilizing stable isotopes of CA and CDCA: respectively [24-^13^C]CA and [24-^13^C]CDCA [56,57,58]. Following oral administration of these isotopes, serum and bile samples are collected over the following days and analyzed for isotope enrichment by gas chromatography-mass spectrometry (GC-MS) [58,59,60]. Fractional turnover rates, pool size and synthesis rates of bile acids can subsequently be calculated as for the radioactive isotope technique. Notably, the measurements from serum samples substantially equaled those from bile. In a similar fashion, the kinetics of secondary bile acids such as deoxycholic acid (DCA) (pool size, input rate) could be determined [61].

In addition, the same research groups described dual isotope procedures for measuring the steady state kinetics of primary and secondary bile acids, using [^2^H] and [^13^C]-labeled bile acids, and serum sampling [58,62,63]. 

A brief mention needs to be made to a novel technique, which has been adopted over the last decades to study cholesterol and bile acid metabolism. This technique relies on mass isotopomer distribution analysis (MIDA) of tracer isotopes as an indirect estimate of isotope enrichment in the precursor pool, and allows the calculation of sterol precursor rates from the direct determination of isotope enrichment in the product. This approach can mainly address the contribution of newly synthesized cholesterol to bile acid synthesis [64].

### 5.2. Studies on the Relative Contributions of the Classical and Alternate Pathways

As reported above, two bile acid synthesis pathways have been described: the classical, occurring in the liver and the alternative one, starting in extra-hepatic tissues [23] and many efforts have been done to explore their relative contribution. Plasma concentrations of 27-hydroxycholesterol and 7α-hydroxycholesterol may be useful to obtain indirect data on this issue, but more information may be obtained by kinetics studies since quantification of metabolic events can be achieved using tracer procedures with stable isotopes [65]. The endogenous rate of production of a metabolite can be calculated from its isotopic enrichment knowing the rate of infusion and plasma concentration of the tested compound under steady state conditions. Therefore, the measurement of the production rate of 27-hydroxycholesterol and 7α-hydroxycholesterol may be used to separately evaluate the two pathways of bile acid synthesis.

Duane and Javitt used for the first time plasma enrichment with isotopes of 27-hydroxycholesterol to explore the alternative pathway by measuring the production rate of such oxysterol. A constant infusion of [^2^H]27-hydroxycholestrol was used to evaluate 27-hydroxycholesterol production rate in normal subjects [26]. Cholesterol 27-hydroxylation pathway averaged approximately 10% of total bile acid synthesis, as evaluated by measuring fecal acidic sterol output. This estimate was based on the rate of 27-hydroxycholesterol production obtained by isotope dilution after 6–10 h of infusion while total bile acid synthesis had been previously calculated from the 72 h fecal acidic sterol output [26]. A nearly complete equilibrium of the infused 27-hydroxycholesterol isotope between serum and liver was shown and the steady state was rapidly achieved. The main limitation of this evaluation refers to the fact that the production rate of 27-hydroxycholesterol evaluated during few hours under fasting conditions was compared with a total bile acid synthesis estimated over a three-day period. Therefore the effects of the alimentation status and of circadian rhythms may render not comparable kinetic data obtained in such different periods of time. In addition the inter-day variability of cholesterol catabolism, mainly through bile acid synthesis, which may occur in normal human subjects as we have observed [66], suggests that the estimation of 10% for the relative contribution of the alternative pathway of bile acid synthesis may be not accurate.

To overcome these limitations a minimally invasive technique, using 2 h infusions of isotopomers of 27-hydroxycholesterol and 7α-hydroxycholesterol was evaluated to simultaneously explore the two major pathways of bile acid synthesis [66]. The steady state was found to be rapidly achieved, however, also this approach has several shortcomings, mainly due to lack of validation of the estimation of the production rate of 7α-hydroxycholesterol. In fact, for 27-hydroxycholesterol, the rate of plasma appearance was previously shown to correspond to its production rate [26] but this may not be the case for 7α-hydroxycholesterol since 7α-hydroxylation is a liver-specific reaction, and the kinetics of the liver-plasma exchange of 7α-hydroxycholesterol are still unknown. In particular, as indicated by experimental observations [66], the isotope ratio for 7α-hydroxycholesterol, reflecting dilution with newly synthesized sterol, in the liver may be lower than in plasma and such systematic overestimation of the isotope ratio may lead to systematic underestimation of the production rate. To obtain an accurate measure of the production rate of 7α-hydroxycholesterol, only invasive techniques to sample liver tissue may be useful. However, even if the rate of plasma appearance of 7α-hydroxycholesterol measured with the isotope dilution techniques may not accurately measure the absolute value of its synthesis, it may be adequate to study modifications in the classical pathway under different conditions.

In conclusion, though many efforts have been made to explore the relative contribution of the classical and alternative pathways of bile acid synthesis using stable isotope dilution techniques, only indirect data are still available. Interestingly, in adult subjects, only for 27-hydroxycholesterol plasma concentration was shown to reflect the production rate [31]. 

The above mentioned procedures can thus provide useful and reliable information on bile acid kinetic parameters, on one side, and on the production rates of 7α-hydroxycholesterol and 27-hydroxycholesterol on the other, the latter being a direct reflection of the efficiency of the classical and alternate biosynthetic pathways. These procedures require analysis by GC-MS, rather sophisticated equipment available only in dedicated laboratory facilities. On the other hand, the absence of safety concerns with the use of stable isotopes makes this approach ideal for human studies, and is therefore believed to completely replace the older radiolabel-based techniques.

## 6. Regulation of Bile Acid Synthesis in Different Situations

Regarding the classical pathway, the rate of transcription, rather than the rate of translation, appears to be the main determinant of the enzymatic activity of CYP7A1 under most physiological circumstances [67]. Reduction in CYP7A1 activity in humans is known to occur with oral administration of CDCA [68], whereas the interruption of the enterohepatic circulation increases its activity thus implying that the bile acid pool size is auto-regulated by the binding of bile acids to the FXR receptor [69]. In case of interruption of the enterohepatic circulation of bile acids, bile acid synthesis is up-regulated and also the receptor for LDL may be up-regulated [44]. Evidence has been provided in different species including humans, that the intestinal flux of bile acids regulates serum levels of intestinal fibroblast growth factors (FGF15-FGF19) that in turn modulate bile acid production in the liver by regulating CYP7A1 activity [70,71].

With regards to the alternative pathway of bile acid synthesis, the regulation of CYP27A1 is relevant mainly for cholesterol reverse transport and protective mechanisms against atherosclerosis. Both 27-hydroxycholesterol and 3β-hydroxy-5-cholestenoic acid, have regulatory functions on cholesterol metabolism, such as the down-regulation of cholesterol synthesis via the sterol regulatory element binding protein (SREBP)/SREBP cleavage-activating protein (SCAP) regulatory pathway [72] and up-regulation of ABC transporter expression, via the liver X receptor (LXR) [73,74]. In cultured cells CYP27A1 was found to be transcriptionally up-regulated by some nuclear receptors, such as retinoid X receptor (RXR) and PPAR gamma [75,76], thus suggesting a possible protective role of these receptors against atherosclerosis.

No kinetics data are available in pediatric settings, but indirect data based on plasma concentrations of oxysterols suggest that the physiologic cholestasis in newborn derives from the immaturity of both pathways of bile acid synthesis [77]. Hepatic cholesterol 7α-hydroxylase activity is absent during fetal life in humans and is up-regulated after birth [44,78]. The classical pathway of bile acid synthesis was shown to increase with age, indicating a complete maturity of the 7α-hydroxylase pathway occurring after the age of 4 years [77]. Also the extra-hepatic pathway of bile acid synthesis is immature in newborn and an efficient up-regulation was observed from the age of 4 years, which was comparable to that found in adult life [77].

With the aid of techniques adopting both radiolabeled and stable isotopes, an enormous body of evidence has been obtained in recent years on the pathophysiology of bile acid synthesis and its regulation in humans *in vivo*, both in spontaneous physiological and pathological conditions and during treatment.

In some instances, the determination of the relative contribution of the two pathways of bile acid synthesis was also possible.

### 6.1. Aging

Aging associates with important metabolic alterations. Evidence with varying experimental approaches *in vivo* utilizing radioactive tracers have shown a progressive reduction of bile acid synthesis with advancing age [79,80]. This is consistent with recent findings [81] confirming a negative correlation among age and hepatic expression of CYP7A1, and serum levels of 7α-hydroxy-4-cholesten-3-one as an indicator of bile acid synthesis and CYP7A1 activity [39,40]. Alterations of hepatic nuclear receptor expression were also described, and correlated with the observed reduction of insulin-like growth factor I (IGF-I), the active metabolite of GH [81]. Altogether, these results appear to suggest a causal relationship between IGF-I levels and bile acid synthesis, which may involve nuclear receptors as co-regulators.

Such changes in bile acid synthesis are likely to account for some metabolic alterations observed with aging, such as increased biliary cholesterol secretion and saturation [79] as well as the increase of serum cholesterol observed in some epidemiological studies [82].

### 6.2. Liver Disease and Cholestasis

Cholesterol metabolism is affected in patients with liver disease for several reasons: (1) cholesterol synthesis occurs within the liver; (2) the large majority of the enzymes involved in bile acid synthesis, which represent the main step for cholesterol elimination, are located within the liver and (3) bile secretion is fundamental for cholesterol elimination. Therefore liver disease is characterized by reduction of cholesterol synthesis, especially in the presence of impaired liver function and, therefore, low plasma concentrations of cholesterol occur in more severe disease [83]. On the other hand, chronic cholestasis is commonly associated with hypercholesterolemia and marked alterations of the enterohepatic circulation of bile acids characterized by decreased biliary secretion with elevated serum levels of bile acids [83,84]. 

In the presence of impaired liver function, bile acid synthesis was found to be reduced [31,55,85] and the reduction was found to be related to the severity of liver disease. Such suppression of bile acid synthesis might reflect a reduction in functioning liver mass, although radioactive isotope studies seem to suggest that CA synthesis is more severely affected than CDCA synthesis [86] (see later).

Finally, data have been obtained with stable isotope administration in a cohort of patients receiving orthotopic liver transplantation, showing no differences in bile acid kinetic parameters and synthetic rates when compared with controls [87].

The role of cholestasis remains to be established, especially in view of the surprising observation that in experimental cholestasis in the rat the activity of 7α-hydroxylase is even increased [88]. However, in humans with obstructive cholestasis evidence is controversial: in patients, bile acid synthesis was reported to be reduced [89,90] even in the absence of changes in CYP7A1 expression [90], whereas another paper reported decreased expression of CYP7A1 associated with changes in FGF-19 expression, normally expressed at the intestine level, being FGF-19 mRNA detected in cholestatic but not in normal liver [91].

Data in animal models have suggested a posttranscriptional inhibition of CYP7A1 activity by oxysterols accumulating in the cholestatic liver [92]. No evidence for increased levels of such sterols has been found in human livers from cholestatic patients [90], however such possibility cannot be completely ruled out. 

Fewer pieces of evidence are available in chronic intrahepatic cholestasis; stable isotope studies showed either no change in kinetic parameters [93] or, in subjects with sclerosing cholangitis, a reduction in the recirculating pool of primary bile acids [94]. 

Kinetic data obtained in patients with liver disease indicate that the classical pathway of bile acid synthesis is affected since a significant reduction of the rate of plasma appearance of 7α-hydroxycholesterol was found in patients with liver disease (chronic hepatitis C or primary biliary cirrhosis) and the defect of the classical pathway was related to the severity of the disease, being the reduction of 7α-hydroxycholesterol plasma appearance more marked in patients with more severe disease [31]. Further studies are needed to separately evaluate the effects of impaired liver function and of cholestasis on the classical pathway of bile acid synthesis.

Finally, kinetic data recently obtained indicate that in patients with liver disease the alternative pathway of bile acid synthesis is completely preserved. In patients with liver disease (chronic hepatitis C or primary biliary cirrhosis), the production rate of 27-hydroxycholesterol was found to be similar to that found in a control group of subjects with normal liver function. Therefore this mechanism which is involved in the reverse transport of cholesterol is completely preserved in patients with liver disease. This may be the reason why neither increase in cardiovascular risk nor accelerated atherosclerosis were found in these hypercholesterolemic patients with liver disease [83,95].

### 6.3. Nutrition and Obesity

The alterations of cholesterol metabolism associated with obesity have been extensively studied in the past, in the attempt to define a causal relationship and possible clues in the pathogenesis of gallstone disease. Radioisotope studies have shown that a linear correlation may exist between body weight and the production rate of primary bile acids [96]. Nonetheless, in obese subjects bile tends to be supersaturated in cholesterol [97,98] and this was ascribed mainly to an increase in cholesterol synthesis in these subjects. The evidence, although scarce, on hepatic CYP7A1 activity *in vitro* [99] also supports the view of an increased bile acid formation in obesity. Increased mobilization of cholesterol from peripheral tissues might also play a role in bile supersaturation.

When, more recently, the production rates of 7α-hydroxycholesterol were assayed by means of a stable isotope infusion, no relevant changes on the classical pathway of bile acid synthesis have been reported in obese patients, when compared to normal weight subjects [11]. 

Preliminary data with stable isotopes also suggest that the production rate of 27-hydroxycholesterol is higher in obese patients compared to healthy subjects [11], thus suggesting that the alternative pathway plays a protective role against atherosclerosis in obese patients, as recently suggested also for hypercholesterolemic patients (see later).

Interestingly, in obese patients, CA synthesis was reduced during a low-calorie diet [100]. This phenomenon has to be taken into account in view of the possible increase in the risk for cholesterol gallstone development, particularly with drastic dieting. 

The effects of modifications of dietary cholesterol intake on bile acid synthesis have been studied with the aid of radiolabeled tracers. Some evidence in humans would support an increase in conditions of increased cholesterol consumption [101,102], whereas other reports failed to describe an augmentation in total bile acid production, measured by classical isotope dilution technique, but showed a relative increase in CDCA synthesis [103]. Interestingly in gallstone patients bile acid synthesis, measured by stable isotope administration, tended to decrease rather than to increase with a cholesterol-rich diet [104]. No direct evidence is available on the molecular mechanisms underlying the possible changes induced by cholesterol feeding; a role for nuclear receptors (LXR α in particular) would seem plausible, even if functional LXR-binding elements are not present in human CYP7A1 promoter [3].

In human subjects fasting was shown to associate with a reduction of CA synthesis, measured by radioisotope dilution technique, in the absence of changes in total bile acid and CA pool size [105].

Animal models of fat-free diet feeding [106] and of total parenteral nutrition [107] showed reduced bile acid synthesis rates, measured by different techniques. Similarly, patients receiving artificial nutrition showed a significant reduction in cholesterol 7α-hydroxylation rates measured by the isotope release approach, compared with control subjects (Bertolotti *et al*, personal observations). In such conditions the reduction in bile acid synthesis seems to be related to a condition of reduced intestinal need for bile acids for absorptive purposes; gastrointestinal hormones and FGF-19 levels might play a relevant role in this regulation. 

The clinical implications of such findings are obvious, considering the high incidence of hepatobiliary complications related to alterations in bile acid metabolism, such as cholelithiasis and cholestasis, during parenteral nutrition. 

Recently, it has been shown by means of stable isotope procedures that total bile acid synthesis decreases both during a low-fat and a high-fat diet, although probably with different mechanisms [108].

Finally, radioisotope kinetics studies in vegetarians showed a slight reduction in CA output, associated with a slight increase in DCA input rate [109].

### 6.4. Metabolic and Endocrine Disorders

The alterations associated with diabetes have been studied in the past. Older studies [110,111] have shown rather inconsistent results, mainly because of heterogeneous patient populations. More recently Brufau *et al*., using a stable isotope approach, have demonstrated an increased synthesis rate of CA (but not of CDCA) and an increased input of DCA in patients with type 2 diabetes [112]. Furthermore, the inverse correlation between synthesis rates and serum FGF-19 levels tended to disappear in diabetic patients. The implications of these findings still have to be defined.

The changes of bile acid synthesis associated with primary hyperlipidemias have also been studied extensively. In familial hypercholesterolemia, a disease characterized by reduced expression of LDL-receptors in the liver, bile acid synthesis does not seem to be affected [113,114] suggesting that receptor-mediated internalization of cholesterol is not essential for conversion to bile acid. The data is consistent with older evidence in patients with phenotype II hyperlipidemia according to Frederickson, where no significant differences in total bile acid synthesis were detected, compared with control, even if a reduced synthesis rate of CA was documented [96].

In forms of hyperlipidemia when hypertriglyceridemia is also present, such as Frederickson type IV hyperlipidemia [96] or, in more recent evidence, familial combined hyperlipidemia [114] and familial hypertriglyceridemia [115], bile acid synthesis is increased. In such conditions increased hepatic production of lipoprotein probably takes place, supporting a coordinate regulation between synthesis of bile acids and lipogenesis; this relationship is still far from being defined in its molecular mechanisms, details but is likely to involve hepatic nuclear receptors (SREBPs in particular). 

When considering the alternate pathway of bile acid synthesis, in hypercholesterolemic patients 27-hydroxycholesterol plasma concentrations were found consistently higher compared with normocholesterolemic patients [31]. Data on the endogenous production rate of 27-hydroxycholesterol, measured by stable isotope infusion, have shown either similar values in normo- and hypercholesterolemic patients [11], or an increase in hydroxylation rates in the latter [116]. At any rate, it can be suggested that higher plasma concentrations are related to increased availability of the substrate rather than to increased enzymatic activity. Whether this might be considered a protective mechanism versus atherogenesis is still debated [27,117].

Alterations of thyroid function profoundly affect cholesterol metabolism, and in particular plasma cholesterol levels, which are typically raised in hypothyroidism and reduced in hyperthyroidism. Therefore, the presence of a correlation between such alterations and modifications of bile acid synthesis has been investigated. Indeed, a study with radioactive tracers in hypothyroid patients did not show any differences in bile acid synthesis rates in comparison with normal controls [118], and a more recent study with stable isotopes showed a rather unexpected slight reduction in synthesis rates in hyperthyroid subjects [119]. These pieces of evidence altogether do not support the view that modifications in cholesterol degradation to bile acids underlie the observed changes in plasma cholesterol. Additional evidence with radioactive tracers however, comparing the two conditions, showed significantly higher rates of bile acid synthesis in hyperthyroidism compared to hypothyroidism [120], consistently with the known metabolic effects of thyroid hormone. 

Cerebrotendinous xanthomatosis (CTX) is a rare autosomal recessive lipid storage disease characterized by non functioning CYP27A1, resulting in a defective alternative pathway, and abnormal deposition of cholestanol and cholesterol in many tissues, including the central nervous system [121,122]. The disease is associated with enhanced risk of premature atherosclerosis and derangement of bile acid metabolism during early life. In these patients data indicating increased production rate of 7α-hydroxycholesterol have been reported thus suggesting an up-regulation of the classical pathway of bile acid synthesis [123,124].

Recently, a new metabolic disorder caused by a homozygous frameshift mutation in the human CYP7A1 gene, that causes the loss of the active site and enzyme function, has been identified in three subjects presenting hyperlipidemia, statin-resistant hypercholesterolemia, premature gallstone disease. In these patients, only data on 27-hydroxycholesterol plasma concentrations are available, suggesting that the alternative pathway of bile acid synthesis is up-regulated [125]. Altogether, the changes observed in the two latter genetic metabolic diseases suggest a mutual regulation of the two pathways of bile acid synthesis.

Alterations in bile acid synthesis might theoretically occur in other disorders of cholesterol metabolism. For instance, Niemann-Pick type C (NPC) disease is a rare and fatal inherited condition, caused by mutations in the NPC1 or (more rarely) in the NPC2 genes. Both gene products are involved in intracellular cholesterol trafficking and ultimately in cholesterol export, so that functional mutations result in abnormal accumulation of cholesterol in most cells of the organism, including vital tissues such as brain, lung and liver [126]. In this condition, alterations of cholesterol degradation to bile acids might be expected; in particular, an increase in the degradation via the different pathways would seem plausible. Indeed, in animal models of NPC disease this does not seem to take place, as shown by Xie *et al.* [127], probably, as the Authors suggest, due to defective access of excess free cholesterol to LXR or to the pools acting as a substrate for CYP7A1 or CYP27A1. The issue is challenging and certainly deserves additional evidence, particularly in humans where no direct data are presently available.

### 6.5. Gallstone Disease

Cholelithiasis is a prevalent disease in the Western world, with a high burden of morbility and health costs. In the past, different attempts have been made to define the presence of single alterations of hepatic cholesterol metabolism which could predispose to increased cholesterol secretion and gallstone formation. In the last few years, alterations of hepatic expression of nuclear receptors has also been investigated in this context [128,129].

On one hand, some pieces of evidence in humans appeared to suggest that a reduction in cholesterol degradation to bile acids could be a predisposing factor for increased biliary cholesterol secretion. This was described in *in vivo* kinetic studies with radioisotope administration [130] and confirmed by *in vitro* assay of reduced CYP7A1 activity [131].

This might apply also to peculiar conditions, as fibrate treatment, where reduced bile acid synthesis was shown to associate with increased cholesterol secretion, in radioisotope kinetic studies [114,132,133] (see later). On the other hand, evidence of increased circulating levels of 7α-hydroxy-4-cholesten-3-one seems to support the existence of a condition of bile acid malabsorption as a factor associated with gallstone prevalence [134].

Additional evidence with radioisotope studies indeed showed no or little changes in bile acid synthesis in gallstone subjects, even if the pool size of primary bile acids tended to be decreased [135,136,137]; the finding was interpreted as an impairment in the storage capacity of the gallbladder. More recent evidence with stable isotope administration also failed to detect changes in bile acid production rate in gallstone patients, with an increase in DCA formation [138].

Cholecystectomy on the other hand was not found to induce significant alterations in CA production rates in older evidence using radioisotope administration [139]; more recent studies with stable isotopes though showed a slight but significant decrease in CA synthesis, and an increase in the fractional conversion of CA to DCA, with no changes in the total formation rate of DCA in consideration of the reduced synthesis of CA [140].

The issue of the possible alterations of cholesterol degradation in gallstone disease is therefore still unsettled [141]. When liver tissue was systematically assayed for specific enzymatic alterations, no definite evidence was shown [142]. Cholelithiasis most likely stands as a multi-factorial conditions, involving a number of genetic, pathophysiological and environmental factors ultimately leading to increased biliary cholesterol saturation which, in the presence of favouring conditions (alterations in gallbladder motility, infection and so on) can trigger nucleation and stone production. In this scenario, bile acid malabsorption might reasonably be considered as an additional risk factor.

### 6.6. Bile Acid Feeding

Bile acids are physiological regulators of biliary lipid metabolism; the effects of the modulation of the bile acid pool on biliary secretion are well known [143], and the effects on different pathways of cholesterol homeostasis have been described as well [144].

The changes induced by treatment with bile acids on bile acid production have been extensively studied. Bile acid administration was shown to reduce bile acid synthesis and CYP7A1 activity. This finding, in addition with the evidence on cholestyramine (see later), strongly supports the view of an inhibitory feedback regulation exerted by bile acids recirculating to the liver. Such inhibitory effect is particularly evident with hydrophobic bile acids, such as CDCA and DCA. On the other hand, treatment with hydrophilic bile acids such as ursodeoxycholic acid (UDCA) generally failed to show any inhibitory effect. This was clearly documented with the assay of CYP7A1 activity in human liver [68] and in studies *in vivo* utilizing different experimental procedures: the classical isotope dilution technique using radioactive bile acids [145,146,147], isotope release of [^3^H]H_2_O from [7α-^3^H]cholesterol [4] and of [^14^C]CO_2_ after administration of [26-^14^C]cholesterol [148], and with the use of stable isotope administration [149].

Similar data were obtained in patients with chronic cholestatic conditions or with obesity, where treatment with UDCA generally failed to show a significant inhibitory effect on bile acid synthesis rates [93,94,100,150].

These data underscore the relevance of the hydrophobic-hydrophilic properties of the recirculating bile acid pool in the control of bile acid synthesis, in agreement with a wide body of evidence in animal models [15,151]. The molecular mechanisms of regulation of bile acid synthesis, and the transcriptional control of the limiting enzyme of the classical biosynthetic pathway, CYP7A1, have been elucidated in recent years and the role of nuclear receptors, including the bile acid receptor FXR, at least in experimental models, has been underlined [5,7,8]. However, the role of nuclear receptors in the physiological regulation of bile acid synthesis and of CYP7A1 expression in humans is still largely unknown [3,81,152]. 

It has to be further recalled that the changes induced on CYP7A1 expression and activity *in vitro* do not always reflect bile acid synthesis. For instance, treatment with DCA was shown to reduce bile acid synthesis measured *in vivo* with radioactive tracers, both by isotope dilution [146] and by isotope release [4], and also to reduce circulating levels of 7α-hydroxy-4-cholesten-3-one, a marker of bile acid synthesis [153]; however, no changes in steady-state mRNA levels of CYP7A1 were observed [154]. These findings might be consistent with the presence of multiple levels of regulation of bile acid synthesis, including transcriptional and non-transcriptional control of CYP7A1 activity. Additional evidence is required in this field.

### 6.7. Drug Treatment

#### 6.7.1. Agents Affecting Lipid Metabolism

Lipid-lowering drugs represent a unique model to investigate the effects of modifications of cholesterol and triglyceride homeostasis on bile acid metabolism.

Bile acid-binding resins like cholestyramine and colesevalam are a powerful tool to obtain parallel changes in bile acid synthesis and plasma cholesterol levels. Increased fecal bile acid loss reduces the inhibitory feedback mechanism on CYP7A1 activity and on bile acid synthesis in the liver [4,49,155,156]. The increased degradation of cholesterol triggers increased HMG-CoA activity, enhanced expression of hepatic LDL receptors and a parallel reduction of plasma LDL-cholesterol levels [4]. Involvement of the SREBP regulatory pathway in this process is likely. A role of FGF-19 in the up-regulation of bile acid synthesis was also described [71].

The findings obtained with cholestyramine were more recently replicated with colesevelam in diabetic patients with the use of stable isotopes [112]. Colesevelam treatment was also shown to increase the contribution of de novo synthesized cholesterol to primary bile acids [157].

Fibric acid derivatives, or fibrates, have long been used in the treatment of hypertriglyceridemia. The hypolipidemic effects of fibrates are largely mediated by the nuclear receptor PPARα, with transcriptional activation of a number of genes involved in lipid and apolipoprotein metabolism. In the past, a body of evidence has described an association between fibrate treatment and increased incidence of cholesterol gallstone disease due to increased biliary cholesterol saturation. This was consistently related to a reduction of bile acid synthesis, as shown with radioisotope release *in vivo*, and of CYP7A1 activity [114,132,133], with a subsequent increase of intracellular availability of free cholesterol recruitable for biliary secretion.

The association between reduced bile acid synthesis and reduced serum levels of triglyceride and apolipoprotein B100 [114] once again supports the hypothesis of coordinate regulation between bile acid and triglyceride/lipoprotein [116].

Statins, competitive inhibitors of HMG-CoA reductase, represent the mainstay of cholesterol-lowering treatment. The transient inhibition of cholesterol synthesis induced by statins triggers SREBP-mediated transcription of enzymes of the cholesterol biosynthetic pathway and of the LDL receptor [158], lowering LDL-cholesterol levels. Statins are a model to study the regulatory role of newly synthesized cholesterol on bile acid metabolism. Distinctly different effects were shown in relation to the different experimental models and settings. Indeed, most evidence currently available in humans *in vivo* and *in vitro* seems to exclude a significant effect of chronic statin treatment on bile acid synthesis and CYP7A1 activity [4,101,102,158,159]. 

Apparently, under stabilized conditions the homeostatic responses triggered by inhibition of cholesterol synthesis can restore an adequate supply of cholesterol for bile acid synthesis. On the other hand, when statin treatment was superimposed to a chronic stimulation of bile acid synthesis with resin treatment, a reduction in cholesterol 7α-hydroxylation rates *in vivo* was disclosed, as measured by isotope release [156]. Similar data were also obtained in bile fistula patients [160]. 

Changes in synthetic rates *in vivo* paralleled changes in plasma lathosterol to cholesterol ratio [156], suggesting that the availability of newly synthesized cholesterol may be critical for bile acid formation in stimulated conditions. An involvement of hepatic nuclear receptors seems likely, even if no direct evidence is available in humans.

Preliminary evidence is also available on the effects of statin treatment on the production of 27-hydroxycholesterol, measured *in vivo* by steady-state stable isotope infusion [116]. Treatment with two different statins, atorvastatin and rosuvastatin, was shown to reduce significantly 27-hydroxylation rates in parallel with a drastic reduction of serum cholesterol. As commented before, the changes observed seem to support the view that plasma cholesterol is the main determinant of 27-hydroxycholesterol formation, even if direct drug effects cannot be completely ruled out.

#### 6.7.2. Other Drugs

Apart from drugs affecting lipid metabolism, a few pieces of evidence are available on the effects induced on bile acid synthesis by different pharmacological treatments. 

Because of the high risk of developing gallstones in women using oral steroid contraceptives, the effects of such drugs was investigated in bile acid kinetic studies [161,162,163]. Production rate and pool size of CA were increased but bile acid secretion was decreased, probably due to decreased recycling. Synthesis rates and pool size of CDCA on the contrary tended to be reduced. However cholesterol secretion significantly increased, accounting for the observed increase in biliary saturation. A significant increase in biliary cholesterol saturation was also observed after administration of conjugated estrogens (Premarin), which associated with reduced synthesis of CDCA [164].

Corticosteroid treatment is extensively utilized in clinical medicine, with a wide array of indications which embrace gastrointestinal diseases as well. Short term treatment with methyl-prednisolone in patients with primary biliary cirrhosis was shown to increase fractional turnover and synthesis rates of CA, assayed by stable isotope dilution technique [165]. Steroid treatment also increased the biliary output of DCA. These findings, although encouraging in terms of changes in biliary cholesterol saturation, warrant caution with respect to the possible hepatotoxic effects of DCA accumulation.

Treatment with the somatostatin analogue octreotide was also studied with stable isotope administration technique; in patients with acromegaly, a typical indication for octreotide treatment, such treatment did not induce any changes in CA synthesis rate, but increased significantly the output of the secondary bile acid DCA [166]. The finding was related to prolongation of large bowel transit, and may play a role as a causal or contributor factor in the pathogenesis of octreotide-related appearance of gallstone disease. Growth hormone treatment, on the other hand, was not found to alter biliary lipid metabolism and bile acid synthesis in healthy adult men [167].

## 7. Concluding Remarks

During the last decade, an extraordinary body of evidence has clarified many aspects of cholesterol catabolism to bile acids: deep insight in the molecular mechanisms of regulation of bile acid synthesis has been reached, and the transcriptional control of CYP7A1 has been defined in great detail along with the regulatory role of nuclear receptors and transcriptional coactivators. Likewise, the role of the alternate pathway of bile acid synthesis and the regulation of CYP27 have been underlined and defined as well. Such progress was made possible, among others, by the widespread implementation of molecular biology techniques.

Nonetheless *in vivo* tracer kinetics studies in the field of cholesterol metabolism and degradation, in humans as well as in animal models, still maintain an exceptional scientific and speculative relevance. On one hand, they allow to overcome the ethical and technical problems concerning invasive procedures, such as liver biopsy, which are needed to perform tissue analysis of enzyme expression and activity. Furthermore, most of the *in vivo* procedures which have been validated so far allow quantitative determination of cholesterol degradation in the unit of time; this represents an obvious advantage towards the assays *in vitro*, and also towards the determination of circulating levels of metabolic precursors, which can certainly be considered a reliable and convenient tool for studies in larger populations, but can provide only comparative, or at the most semi-quantitative, data. The information obtained in tracer studies can be utilized for balance studies, or simulations, regarding cholesterol homeostasis.

It must also be recalled that evidence obtained from tissue analysis *in vitro* does not always reflect the findings obtained in *in vivo* studies: this usually represents a challenging situation, and sometimes allows interesting pathophysiological speculations (e.g., the presence of post-transcriptional levels of control, competitive inhibition that cannot be detected by *in vitro* assays, and so on). Tracer studies *in vivo*, therefore, can be considered a valuable complement, rather than an alternative, to the analyses on tissues *in vitro*. 

Nowadays studies with non-radioactive stable isotopes have virtually replaced the older approaches with radiolabels, and have become the gold standard for kinetic studies *in vivo* due to the absence of radiological invasiveness. Once again, widespread use of GS-MS expertise will likely make those approaches more largely available to the scientific community.

Tracer kinetic studies can prove as exceptional tools for the investigation of sterol metabolism in the future, as they have been in the past; their speculative and translational implications will hopefully help to disclose new pathophysiological knowledge, and maybe novel management strategies, in the field of cholesterol accumulation conditions and their important clinical consequences.
